# The Alterations in the Expression and Function of P-Glycoprotein in Vitamin A-Deficient Rats as well as the Effect of Drug Disposition *in Vivo*

**DOI:** 10.3390/molecules21010046

**Published:** 2015-12-29

**Authors:** Yubang Wang, Heng Qin, Chengxiang Zhang, Fei Huan, Ting Yan, Lulu Zhang

**Affiliations:** 1 The Key Laboratory of Modern Toxicology of Ministry of Education, Department of Toxicology, School of Public Health, Nanjing Medical University, Nanjing 211166, Jiangsu, China; wyb@njmu.edu.cn (Y.W.); qh@njmu.edu.cn (H.Q.); zcx@njmu.edu.cn (C.Z.); huanfei@njmu.edu.cn (F.H.); yanting@njmu.edu.cn (T.Y.); 2 The Center for Hygienic Analysis and Detection, Nanjing Medical University, Nanjing 211166, Jiangsu, China

**Keywords:** vitamin A deficiency, P-glycoprotein, rhodamine 123, vincristine

## Abstract

This study was aimed to investigate whether vitamin A deficiency could alter P-GP expression and function in tissues of rats and whether such effects affected the drug distribution *in vivo* of vitamin A-deficient rats. We induced vitamin A-deficient rats by giving them a vitamin A-free diet for 12 weeks. Then, Abcb1/P-GP expression was evaluated by qRT-PCR and Western blot. qRT-PCR analysis revealed that Abcb1a mRNA levels were increased in hippocampus and liver. In kidney, it only showed an upward trend. Abcb1b mRNA levels were increased in hippocampus, but decreased in cerebral cortex, liver and kidney. Western blot results were in good accordance with the alterations of Abcb1b mRNA levels. P-GP function was investigated through tissue distribution and body fluid excretion of rhodamine 123 (Rho123), and the results proclaimed that P-GP activities were also in good accordance with P-GP expression in cerebral cortex, liver and kidney. The change of drug distribution was also investigated through the tissue distribution of vincristine, and the results showed a significantly upward trend in all indicated tissues of vitamin A-deficient rats. In conclusion, vitamin A deficiency may alter Abcb1/P-GP expression and function in rat tissues, and the alterations may increase drug activity/toxicity through the increase of tissue accumulation.

## 1. Introduction

Vitamin A deficiency is a metabolic disorder caused by lack of vitamin A *in vivo*. It may cause various diseases, such as night blindness, retarded growth, malnutrition and even death. Furthermore, vitamin A metabolic disorder may cause cytochromeP450s (CYP450s) and efflux transporters alterations [1,2,3] and then alter drug distribution in tissues, accompanied by affecting the toxicity and activity of drugs.

The efflux transporters mainly belong to the ATP-binding cassette (ABC) super-families, and substances involved in their transport include amino acids, proteins, polypeptides, metal ions and drugs. It was found that there were forty-eight ABC transporter genes in humans [4], and at least eight ABC transporters participated in drug efflux transport, comprising P-glycoprotein (P-GP, ABCB1), breast cancer resistance protein (BCRP, ABCG2) and multidrug resistance-associated proteins (MRPs1-6, ABCC1-6). P-GP has abundant structural types of substrates and acts as a drug efflux pump, exporting vinca alkaloids, cyclosporine, calcium channel blockers, antibiotics, immunosuppressants, lipid and steroids [5,6]. P-GP is expressed in various tissues, such as brain, lung, liver, kidney, gastrointestinal tract, skin and muscle [5,7], and is considered to be an important component of the blood-brain barrier (BBB), the blood-placenta barrier, the blood-testis barrier and other biological barriers *in vivo* [8,9]. Therefore, P-GP may be one of the important factors that restrict drug distribution.

The occurrence and development of many diseases may be associated with metabolic disorders of vitamin A. Additionally, it is also known that P-GP expression and function may be affected by the above pathophysiological conditions, such as cancer, chronic renal failure, nonalcoholic fatty liver disease and diabetes [10,11,12,13]. Several studies showed that vitamin A level may regulate the expression of CYP450s. Shiyang Chen *et al.* found that retinol might regulate CYP3A expression by activating the RXR/CAR pathway [2]. Kun Wang *et al*. also found that in HepG-2 and Caco-2 cells, retinol might induce CYP3A expression through the RXR/VDR pathway [3]. It is well known that CYP450s and efflux transporters have the same upstream regulatory pathway, and ABC transporters’ expression may be also regulated by PXR (pregnane X receptor), RXR, CAR, FXR (farnesoid X receptor) and LXR (liver X receptor) [14,15]. This indicated that vitamin A level may affect P-GP expression by a similar pathway as the CYP450s. Additionally, vitamin A metabolic disorder may cause abnormal energy metabolism, and the efflux transport of most substances is a process of energy dissipation [16,17]. This indicated that vitamin A may also affect P-GP expression and function through an energy metabolic pathway. As most drugs acted as a P-GP substrate, this suggested that the alterations of P-GP expression and function in rats under vitamin A deficiency may affect the tissue distribution of drugs and then alter drug toxicity and activity. All of these studies give the hypothesis that vitamin A deficiency may alter P-GP expression and function; besides, the alterations may cause a change to the drug distribution.

The purpose of the study was to verify our hypothesis using vitamin A-deficient rats as an animal model. The levels of Abcb1/P-GP in the indicated tissues were estimated by qRT-PCR analysis and Western blot, respectively. The P-GP function was assessed by measuring the distribution and excretion of rhodamine 123 (Rho123). Additionally, the drug distribution was investigated through the tissue distribution of vincristine.

## 2. Results and Discussion

### 2.1. Physiological and Biochemical Parameters of Experimental Rats

The physiological and biochemical parameters of experimental rats were measured and are listed in Table 1. Compared to the age-matched normal control rats, lower total food intake, as well as a lower level of vitamin A were found in vitamin A-deficient rats (*p* < 0.01).

**Table 1:** The physiological and biochemical parameters of experimental rats.

Parameters	Control	Vitamin A Deficiency
Body weight (g)	504 ± 23	486 ± 38
Total food intake (g)	2274 ± 216	1999 ± 270 **
Vitamin A level in liver (µg/g)	10.6 ± 1.4	2.4 ± 0.8 **
Vitamin A level in serum (µg/mL)	13.5 ± 2.3	3.1 ± 0.7 **

Data are shown as the mean ± SD of thirty rats. ** *p* < 0.01 *vs.* control values using ANOVA statistics followed by the Student–Newman–Keuls multiple comparison *post hoc* test.

### 2.2. Level of Abcb1 mRNA

**Figure 1:**
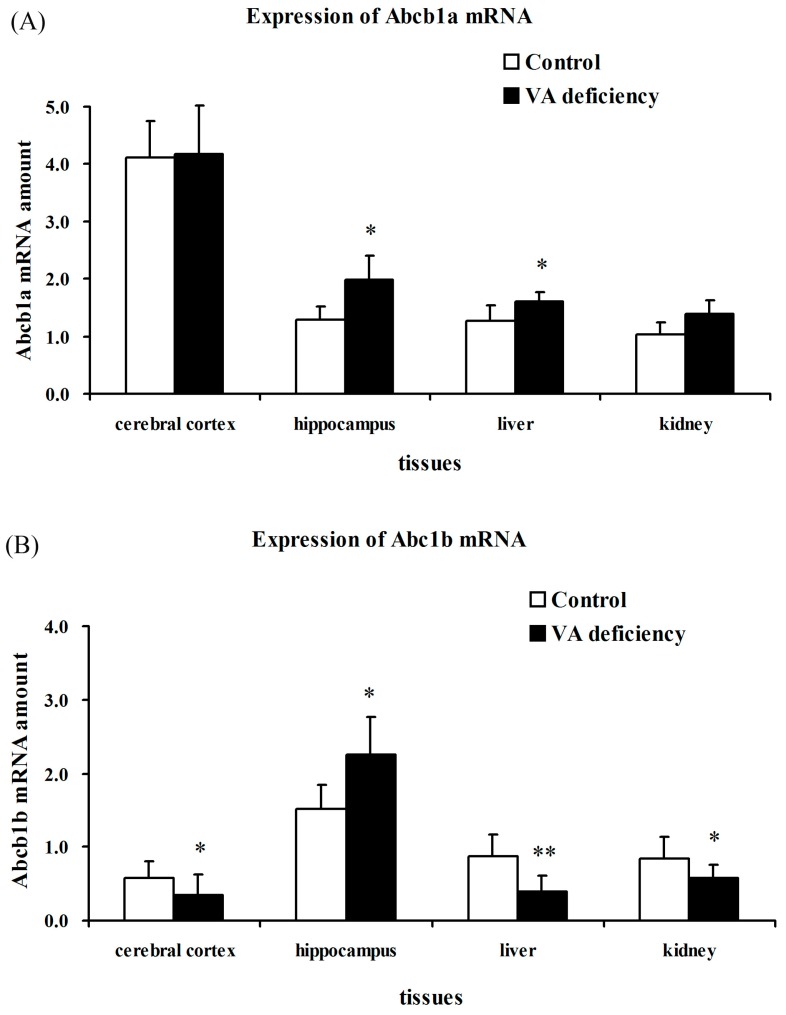
Effects of vitamin A (VA) deficiency on Abcb1a/1b mRNA levels in cerebral cortex, hippocampus, liver and kidney of experimental rats. Relative stain intensity for Abcb1a mRNA levels (**A**) and Abcb1b mRNA levels (**B**) in indicated tissues of experimental rats are presented. qRT-PCR was used to measure levels of Abcb1a/1b mRNA in indicated tissues, and each datum represents the mean ± SD of four rats. * *p* < 0.05; ** *p* < 0.01 *vs.* control values using ANOVA statistics followed by the Student–Newman–Keuls multiple comparison *post hoc* test.

In rodents, Abcb1/P-GP exists as two functional isoforms, which are encoded by two different genes: Abcb1a and Abcb1b. qRT-PCR was used to measure the levels of Abcb1a/1b mRNA in indicated tissues. Their mRNA levels were normalized for the cycle threshold values for the housekeeping gene, β-actin. The results showed that Abcb1a mRNA levels were significantly upregulated in hippocampus and liver of vitamin A-deficient rats compared to the age-matched normal control rats (*p* < 0.05). However, it only showed an upward trend in kidney, while it showed no change in cerebral cortex by vitamin A deficiency. Abcb1b mRNA levels also increased in hippocampus (*p* < 0.05), but decreased in cerebral cortex (*p* < 0.05), liver (*p* < 0.01) and kidney (*p* < 0.05) (Figure 1).

### 2.3. Expression of P-GP Protein

**Figure 2:**
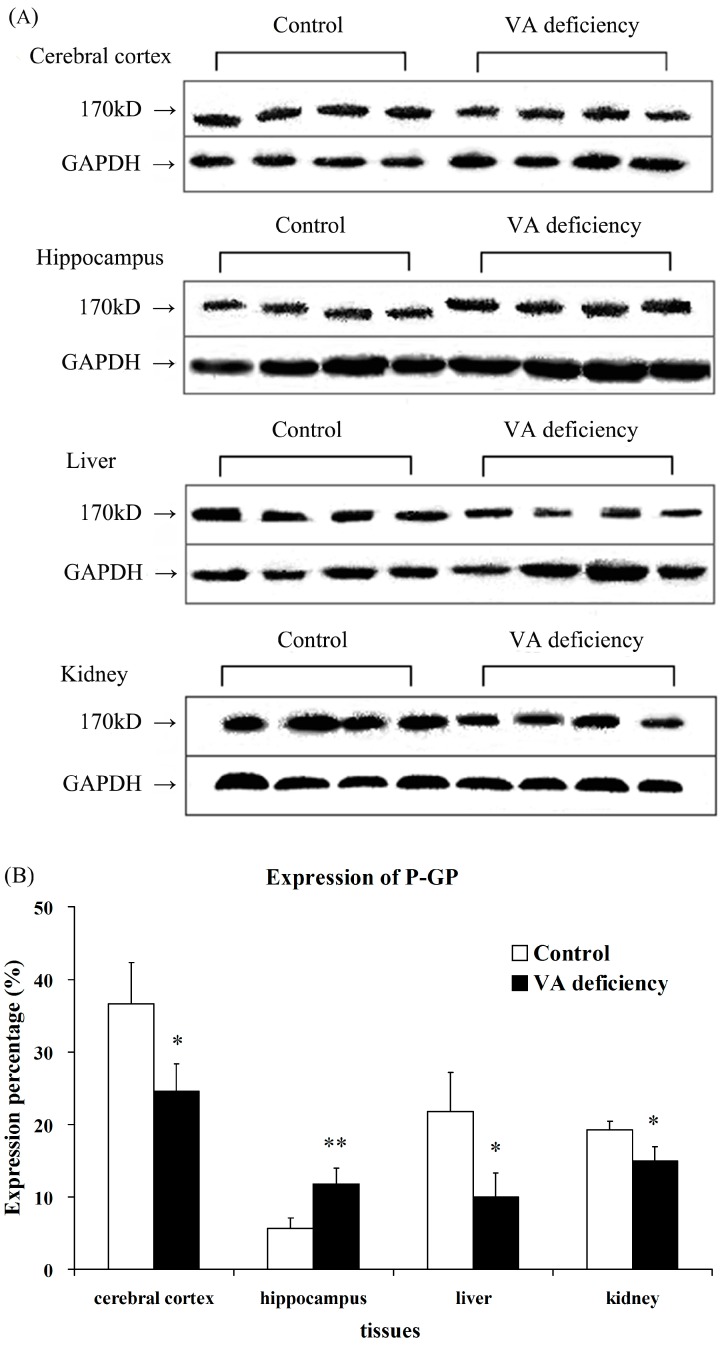
Effects of vitamin A (VA) deficiency on protein levels of P-GP in cerebral cortex, hippocampus, liver and kidney of experimental rats. Representative Western blot stains of P-GP (**A**) and the ratios of relative stain intensity for P-GP (**B**) in indicated tissues of experimental rats are described. Each band corresponding to 170 kDa was observed. Western blot was used to investigate the P-GP protein expression in indicated tissues, and each datum represents the mean ± SD of four rats. * *p* < 0.05; ** *p* < 0.01 *vs.* control values using ANOVA statistics followed by the Student–Newman–Keuls multiple comparison *post hoc* test.

Western blot was used to investigate the P-GP protein expression in indicated tissues. The results revealed a band of 170 kDa, corresponding to P-GP. It was found that P-GP expression in vitamin A-deficient rats was markedly upregulated in hippocampus (*p* < 0.01) compared to age-matched control rats. However, the P-GP expression was significantly downregulated in cerebral cortex, liver and kidney (*p* < 0.05) compared to age-matched control rats (Figure 2).

### 2.4. Tissue Distribution of Rho123

To investigate whether the alterations of P-GP expression induced by vitamin A deficiency affected P-GP functional activity, the distribution of Rho123, a typical substrate of P-GP, in the previously-mentioned tissues was measured 45 min after an i.v. dose of Rho123. The tissue-to-plasma concentration ratio was calculated as an index of P-GP functional activity. The results (Table 2) indicated that vitamin A deficiency increased the concentrations of Rho123 in cerebral cortex, liver and kidney, leading to a higher tissue-to-plasma concentration ratio (*p* < 0.05). However, it only showed an increasing trend in hippocampus.

**Table 2:** Effects of vitamin A deficiency on rhodamine 123 (Rho123) distribution in cerebral cortex, hippocampus, liver and kidney.

Parameters	Control	Vitamin A Deficiency
Plasma (ng·mL^−1^)	42.74 ± 3.51	35.64 ± 1.78 *
Cerebral cortex (ng·g^−1^)	3.71 ± 0.14	8.35 ± 0.97 *
*K*_p_ (cerebral cortex, mL·g^−1^)	0.09 ± 0.04	0.23 ± 0.07 *
Hippocampus (ng·g^−1^)	4.37 ± 0.86	5.17 ± 1.79
*K*_p_ (hippocampus, mL·g^−1^)	0.10 ± 0.03	0.14 ± 0.05
Liver (ng·g^−1^)	49.40 ± 13.42	72.06 ± 17.31 *
*K*_p_ (liver, mL·g^−1^)	1.16 ± 0.26	2.02 ± 0.71 *
Kidney (ng·g^−1^)	1065.08 ± 259.67	1268.43 ± 314.57
*K*_p_ (kidney, mL·g^−1^)	24.92 ± 6.46	35.59 ± 9.74 *

The concentrations of Rho123 in plasma and tissues were measured at 45 min following administration of Rho123 (0.2 mg·kg^−1^, i.v.). Each datum represents the mean ± SD of five rats. Brain, liver and kidney were represented by ng·g^−1^ tissue. *K*_p_ represents the tissue-to-plasma concentration ratio. * *p* < 0.05 *vs.* control values using ANOVA statistics followed by the Student–Newman–Keuls multiple comparison *post hoc* test.

### 2.5. Biliary Excretion and Urinary Excretion of Rho123

**Figure 3:**
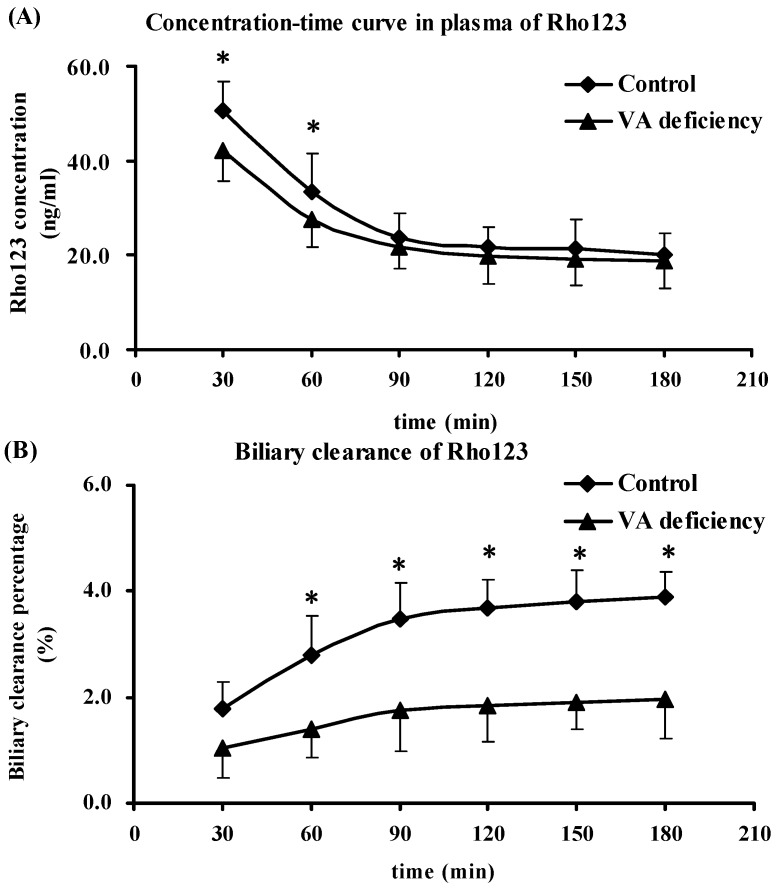

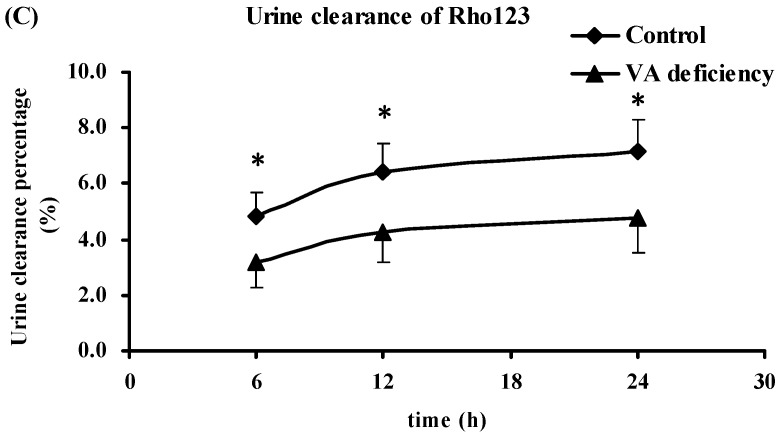
Effects of vitamin A (VA) deficiency on plasma AUC_0–180min_ (**A**) and accumulated excretion percentages of Rho123 in biliary excretion (**B**) and urine (**C**). Biliary excretion and urinary excretion of Rho123 were used to investigate the functional activities of P-GP in liver and kidney, and the plasma AUC_0–180min_ was also estimated. Each datum represents the mean ± SD of five rats. * *p* < 0.05 *vs*. the control values using ANOVA statistics followed by the Student–Newman–Keuls multiple comparison *post hoc* test.

The functional activities of P-GP in liver and kidney were further evaluated using biliary excretion and urinary excretion of Rho123, respectively. The plasma AUC_0–180min_ was also estimated. The results showed (Figure 3) that plasma AUC_0–180min_ was significantly decreased compared to the age-matched control rats (*p* < 0.05), and it showed a clear decrease in biliary excretion and urinary excretion of Rho123 in vitamin A-deficient rats (*p* < 0.05). The results indicated that vitamin A deficiency may decrease P-GP function in liver and kidney, which was in agreement with the findings in P-GP expression.

### 2.6. Tissue Distribution of Vincristine

To investigate whether the alterations of P-GP expression and functional activity induced by vitamin A deficiency affected P-GP substrate distribution *in vivo*, the distribution of vincristine (VCR), a typical anti-tumor drug, as well as P-GP substrate, in indicated tissues, was measured at 30 min after an i.v. dose of VCR. The tissue-to-plasma concentration ratio was calculated as an index of VCR distribution. The results (Table 3) indicated that vitamin A deficiency increased the concentrations of VCR in cerebral cortex (*p* < 0.05), hippocampus (*p* < 0.05), liver (*p* < 0.01) and kidney (*p* < 0.05), leading to a higher tissue-to-plasma concentration ratio.

**Table 3:** Effects of vitamin A deficiency on the vincristine (VCR) distribution in cerebral cortex, hippocampus, liver and kidney.

Parameters	Control	Vitamin A Deficiency
Plasma (ng·mL^−1^)	39.47 ± 9.51	30.41 ± 4.78 *
Cerebral cortex (ng·g^−1^)	13.02 ± 3.38	34.97 ± 5.97 *
*K*_p_ (cerebral cortex, mL·g^−1^)	0.33 ± 0.67	1.15 ± 0.32 *
Hippocampus (ng·g^−1^)	10.65 ± 3.99	15.51 ± 4.06 *
*K*_p_ (hippocampus, mL·g^−1^)	0.27 ± 0.43	0.51 ± 0.37 *
Liver (ng·g^−1^)	574.68 ± 38.42	950.92 ± 35.25 **
*K*_p_ (liver, mL·g^−1^)	14.56 ± 3.51	31.27 ± 6.21 **
Kidney (ng·g^−1^)	1640.37 ± 59.67	1714.82 ± 41.57 *
*K*_p_ (kidney, mL·g^−1^)	41.56 ± 4.64	56.59 ± 3.31 **

The concentrations of VCR in plasma and tissues were measured at 30 min following administration of VCR (4.0 mg·kg^−1^, i.v.). Each datum represents the mean ± SD of five rats. Brain, liver and kidney were represented by ng·g^−1^ tissue. *K*_p_ represents the tissue-to-plasma concentration ratio. * *p* < 0.05; ** *p* < 0.01 *vs.* control values using ANOVA statistics followed by the Student–Newman–Keuls multiple comparison *post hoc* test.

## 3. Materials and Methods

### 3.1. Animals

Male Sprague-Dawley weanling rats, weighing 70–80 g, were purchased from Sino-British Sippr/BK Laboratory Animal Ltd. (Shanghai, China). The rats were housed under controlled environmental conditions (temperature, 25 ± 1 °C; humidity, 55% ± 5%) and kept under a 12-h light/dark cycle; commercial food and water were freely available. The animal protocols used in this work were evaluated and approved by the Animal Use and Ethic Committee of Jiangsu Science and Technology Office (China), Nanjing Medical University. They are in accordance with Federation for European Laboratory Animal Science Assocations (FELASA) guidelines and the National Law for Laboratory Animal Experimentation. Every effort was made to minimize stress to the animals.

### 3.2. Vitamin A Deficiency Model

Vitamin A deficiency was induced in rats by a vitamin A-free diet for 12 weeks. The age-matched normal control rats were fed with a normal diet. The components of the diet are shown in Table 4. Vitamin A deficiency was confirmed by visually, as well as the vitamin A level in rat liver and blood serum. As described previously, vitamin A-deficient rats will be established by rough, none-glossy fur accompanied by photophobia and a significantly lower vitamin A level than the control rats [18]. By the end of 12 weeks, rats from each group were randomly chosen for measuring Abcb1 mRNA, P-GP protein levels, P-GP function, as well as drug distribution in rat cerebral cortex, hippocampus, liver and kidney following the determination of vitamin A level.

**Table 4:** The composition of the rat diets (%).

Composition	Control Diet	Vitamin A-Free Diet
Casein	20%	20%
Gelatinized-starch	44.5%	44.5%
Sucrose	22%	22%
Corn oil	5%	5%
Cellulose	2%	2%
Mineral mixture	5%	5%
Vitamin mixture	1%	—
Vitamin A-free vitamin mixture	—	1%
methionine	0.3%	0.3%
Choline bitartrate	0.2%	0.2%

### 3.3. qRT-PCR Analysis

qRT-PCR analysis was used to measure Abcb1 mRNA levels in rat cerebral cortex, hippocampus, liver and kidney. The experimental rats were sacrificed under ether anesthesia, and the indicated tissues were quickly obtained. Each tissue, weighing 50 mg, was homogenized under ice-cold condition. The qRT-PCR procedure was conducted as previously described [13,19]. Briefly, 2 μg of total RNA from each original sample were converted into cDNA for each individual qRT-PCR assay in a 38-cycle three-step PCR using the ABI Prism 7000 thermocycler. PCR primer sequences are shown in Table 5. Amplification was performed in 20 μL reaction mixture: 2.0 μL of 10× PCR buffer, 2.0 μL of 25 mM MgCl_2_, 0.4 μL of 10 mM deoxyribonucleoside triphosphate, 250 nM of the appropriate forward and reverse primers (Abcb1a/1b and β-actin) and SYBR green I (Molecular Probes, OR, USA). For normalization of the gene levels, β-actin was used to correct minor variations in the input RNA amount or inefficiencies of the reverse transcription. The results were calculated according to Applied-Biosystems.

**Table 5:** Primer characteristics of Abcb1a, Abcb1b and β-actin.

Gene	GeneBank	Amplicon (bp)	Sequence Forward	Sequence Reverse
Abcb1a	AF257746	351	5′-GCCCTGTTCTTGGACTGT-3′	5′-GGCCGTGATAGCTTTCTT-3′
Abcb1b	AY082609	351	5′-GCCCATCCTGTTTGACTG-3′	5′-CGCTTCCTGGACGACCTT-3′
β-actin	NM_007393.3	365	5′-TGACGTGGACATCCGCAAAG-3′	5′-CTGGAAGGTGGACAGCGAGG-3′

### 3.4. Western Blot Assay

The Western blot assay was used for assessing P-GP protein expression in rat cerebral cortex, hippocampus, liver and kidney according to the method previously described [13,20]. The experimental rats were sacrificed under ether anesthesia, and the indicated tissues were quickly obtained. Each tissue, weighing 100 mg, was homogenated and lysed in lysis buffer containing 10 mM Tris-HCl (pH 7.5), 1 mM EGTA, 1 mM MgCl_2_, 1 mM mercaptoethanol, 1% glycerol and protease inhibitor cocktail (1 mM dithiothreitol, 2 mM phenylmethylsulphonylfluoride). The lysate was incubated on ice for 30 min and centrifuged at 13,000× *g* for 10 min at 4 °C. The supernatant was obtained as membrane fractions for Western blot. The protein concentration in the solution was measured by the Bio-Rad Protein Assay. An aliquot of tissue sample was diluted with a volume of 4× sodium dodecyl sulfate (SDS) sample buffer containing 0.1 M Tris-HCl (pH 6.8), 4% SDS, 200 mM dithiothreitol (DTT), 20% glycerol and 0.2% bromophenol blue. Proteins (25 μg per lane) were separated by electrophoresis on 8% SDS-polyacrylamide gel. After electrophoresis, the proteins were electrophoretically transferred to a nitrocellulose membrane. The membrane was blocked in phosphate-buffered saline (PBS) containing 0.1% Tween-20 (PBST) and 5% dried skim milk for 60 min at room temperature and washed three times for 15 min in PBST. Then, the membrane was incubated with the primary monoclonal antibody C219, diluted 500-fold in PBST overnight at 4 °C. After removal of the primary antibody, the membrane was washed with PBST, and then, it was incubated in the appropriate HRP-conjugated goat anti-mouse secondary antibody at room temperature for another 1 h and washed again three times in PBST. The transferred proteins were incubated with enhanced chemiluminescence (ECL) substrate solution for 5 min according to the manufacturer’s instructions and visualized with autoradiography X-film. The relative levels were quantified densitometrically by using the Quantity One software (Bio-Rad Laboratories, Richmond, CA, USA) and calculated according to the reference bands of glyceraldehyde phosphate dehydrogenase (GAPDH).

### 3.5. Tissue Distribution of Rho123

To elucidate the alterations of P-GP function in rat cerebral cortex, hippocampus, liver and kidney, Rho123, a typical P-GP substrate, was intravenously administered to the experimental rats at a 0.2 mg·kg^−1^ dose [13,20]. At 45 min after administration, the rats were sacrificed under light ether anesthesia, and then, the indicated tissues and blood samples were obtained for measuring concentrations of Rho123.

### 3.6. Biliary Excretion and Urinary Excretion of Rho123

To further confirm the alterations of P-GP function in rat liver and kidney, biliary excretion and urinary excretion were investigated. On the experiment day, five rats from each group were randomly chosen and individually housed in the metabolic cage after administering Rho123 (0.2 mg·kg^−1^, i.v.). Urine was collected during 0–6 h, 0–12 h and 0–24 h [13,20].

To evaluate the biliary excretion of Rho123, bile duct intubation was performed as described previously [13,21]. The experimental rats were anesthetized by an intraperitoneal injection of pentobarbital sodium (45 mg·kg^−1^). The bile duct was cannulated with a polyethylene tubing (PE10). The abdomen was covered with saline-saturated gauze to maintain the moisture. Rho123 was given to rats via tail vein at a 0.2 mg·kg^−1^ dose. Then, bile samples were collected every 30 min up to 180 min. At the same time, blood was collected via the jugular vein, and the plasma was obtained by centrifugation (3500 rpm × 10 min). The plasma, bile and urine samples were stored at −20 °C for analysis. The accumulated excretion percentages were calculated, and the area under curve of plasma concentration (AUC_0–180min_) was estimated using the linear trapezoidal rule.

### 3.7. Tissue Distribution of Vincristine

To elucidate the effect of alterations of P-GP function and expression in rat cerebral cortex, hippocampus, liver and kidney on drug distribution *in vivo*, vincristine, a typical anti-tumor drug, as well as P-GP substrate were intravenously administered to the experimental rats at a 4 mg·kg^−^^1^ dose. At 30 min after administration, the rats were sacrificed under light ether anesthesia, and then, the indicated tissues and blood samples were obtained for measuring the concentrations of vincristine.

### 3.8. Sample Assay

The concentrations of vitamin A in rat liver and serum were measured by a high-performance liquid chromatography (HPLC) according to the method previously described [18]. The HPLC system consisted of an Agilent 1260 Infinity System (Agilent, Palo Alto, CA, USA), a ZORBAX Eclipse Plus C18, 150 mm × 4.6 mm i.d., 5-μm particle size column (Agilent), and a diode array detector (DAD-G4212B) set at a wave-length of 325 nm. The rat liver was homogenized in chloroform/methanol (2:1, *v*/*v*) solution, and then, the homogenate was added up to 50 mL with the above solution, besides, a 20% volume of water was added to the homogenate and left overnight. The upper layer was moved, and 40 mL methanol were added to the lower layer; then, the extracted solution was dried; after that, 3% pyrogallol and sodium hydroxide were added, and the solution was incubated at 70 °C for 30 min. Afterwards, the solution was extracted with hexane three times. The extracts were dried and dissolved with methanol. Twenty microliters of the supernatant were injected into the HPLC system. The mobile phase consisted of methanol and water (92:8, *v*/*v*), and the flow rate was set to be 1.0 mL·min^−1^.

The concentrations of Rho123 in tissues, bile, urine and plasma were measured by HPLC according to the method previously described [13,20]. The HPLC system consisted of an Agilent 1260 Infinity System (Agilent), a ZORBAX Eclipse Plus C18, 150 mm × 4.6 mm i.d., 5-μm particle size column (Agilent), and a fluorescence detector (RID-G1321B) set at an excitation wave-length of 485 nm and an emission wavelength of 546 nm. Tissues were homogenized with physiological saline, and 100 µL of plasma, bile, urine and tissue homogenate were vortexed with 300 µL methanol for 10 min, respectively. After centrifugation (20,000 rpm × 10 min), 200 µL of the supernatant were taken out and centrifuged again. Twenty microliters of the supernatant were injected into the HPLC system. The mobile phase consisted of 0.1% glacial acetic acid (pH 4.0) and acetonitrile (7:3, *v*/*v*), and the flow rate was set to be 1.0 mL·min^−1^.

The concentrations of vincristine in tissues and plasma were measured by an ultra-performance liquid chromatography-electrospray ionization-tandem mass spectrometry (UPLC-ESI-MS/MS) according to the method previously described [22,23,24]. The UPLC-MS/MS system consisted of a Waters I-class System (Waters, Milford, MA, USA), HSS T3 C18, 50 mm × 2.1 mm i.d., 1.8-μm particle size column (Waters) and electro spray ion source (ESI) (Waters). Tissues were homogenized with physiological saline, and 300 µL of plasma and tissue homogenate were vortexed with 30 µL methanol, respectively. Then, 40 µL 0.5 M phosphoric acid solution and 4 mL chloroform were added to the homogenate and vortexed again for 1 min; after centrifugation (10,000 rpm × 10 min), the lower layer was obtained and dried with N_2_. The residue was dissolved with the mobile phase. One microliter of the supernatant was injected into the UPLC-MS/MS system. The mobile phase consisted of 15 nM ammonium acetate containing 0.02% formic acid and methanol (35:65, *v*/*v*), and the flow rate was set to be 0.2 mL·min^−1^. The quantification of vincristine was based on the Multiple Reaction Monitoring (MRM) mode.

### 3.9. Data Analysis

Results were expressed as the mean ± standard deviation (SD). The overall differences among groups were determined by one-way of analysis of variance (ANOVA). If the analysis was significant, the differences between groups were estimated using the Student–Newman–Keuls multiple comparison *post hoc* test. A *p*-value of less than 0.05 indicated a significant difference.

### 3.10. Materials

The vitamin A standard reference was purchased from National Institutes for Food and Drug Control (Beijing, China). The vincristine (VCR) injection was provided by Jiangsu Province Hospital. The vitamin A-free vitamin mixture was bought from Oriental Yeast Co., Ltd. (Chiba, Japan). Primers for the Abcb1a/1b and β-actin genes used in qRT-PCR analysis, as well as P-glycoprotein monoclonal antibody were provided by Mogene Bio-technologies, Inc. (Nanjing, China). The Bio-Rad Protein Assay was provided by Bio-Rad Laboratories; enhanced chemiluminescence (ECL) substrate solution was bought from Cell Signaling Technology, Inc. (Palo Alto, CA, USA); protease inhibitor cocktail, pentobarbital and rhodamine 123 (Rho123) were all purchased from Sigma Chemical Co. (St. Louis, MO, USA). All other reagents were commercially available and were of analytical grade. Both Rho123 and pentobarbital were dissolved in physiological saline before use.

## 4. Conclusions

The present study was undertaken to investigate the effect of vitamin A deficiency on P-GP expression and function in cerebral cortex, hippocampus, liver and kidney. The results clearly demonstrated that vitamin A deficiency not only altered P-GP expression and function in the indicated tissues, but also affected P-GP substrate distributions *in vivo*.

qRT-PCR and Western blot analysis (Figure 1 and Figure 2) showed that P-GP expression was in good accordance with Abcb1 mRNA levels in most of the indicated tissues. Surprisingly, the alterations of Abcb1a and Abcb1b mRNA levels in liver were in different directions, and P-GP expression was more consistent with the Abcb1b mRNA level. The meaning and mechanism for different alterations in either Abcb1 isoform remains unknown, although it has been proposed that alterations in Abcb1b mRNA levels may compensate for the changes of Abcb1a [25]. Piet Borst and Alfred H. Schinkel generated knockouts of the three P-glycoprotein genes of mice, the Mdr1a, Mdr1b and Mdr2, and showed that loss of Mdr1a (Abcb1a) had a profound effect on the tissue distribution and especially the brain accumulation of a range of drugs frequently used in humans. All drugs were shown to be excellent substrates of the murine Abcb1a and its human counterpart, MDR1 [26]. While in addition to their common function as a drug efflux transporter, the Abcb1 isoform appears to have its specific function: the Abcb1a was reported to regulate cell volume by influencing swelling, activating chloride currents via a protein kinase C-sensitive phosphorylation site on P-GP [27], while the Abcb1b was involved in apoptotic mechanisms [28] and cellular stress [29,30]. However, these points are still controversial.

Rho123, a typical substrate of P-GP, has been widely used as an indicator of P-GP-mediated transport in *in vitro* and *in vivo* studies [31,32,33,34]. In the present study, Rho123 also served as a marker for evaluating P-GP function. The tissue-to-plasma concentration ratio, biliary excretion and urinary excretion of Rho123 were used for assaying P-GP activities in cerebral cortex, hippocampus, liver and kidney, respectively. The results (Table 2, Figure 3) demonstrated that under the vitamin A deficiency condition, significant increases in tissue-to-plasma concentration ratios in the indicated tissues and lowered biliary and urinary excretion in liver and kidney of Rho123 were found. The higher tissue-to-plasma concentration ratios were postulated to be due to decreased P-GP expression in tissues, resulting in an increased accumulation of its substrate in tissues. The decrease of Rho123 exposure in plasma may result from the increase of P450s or other metabolism-related enzymes [35], and the increased levels of Rho123 in tissues may result from the P-GP function impairment or tissue barrier integrity damage. The increases in brain-to-plasma concentration ratios indicated that the P-GP function might be impaired in the blood brain barrier (BBB). The alteration in cell-cell contact [36] and disruption of tight junction [37,38] in BBB may be the reasons for the increase in the tissue-to-plasma concentration ratio in brain. Lowered biliary and urinary excretion of Rho123 in vitamin A-deficient rats may partly explain the increase in liver-to-plasma and kidney-to-plasma concentration ratios, indicating the impairment of P-GP function in liver and kidney. This indicated that there were some factors causing a decline of the binding affinity of P-GP to its substrate or to ATP, which contributed to the lowered efflux activity of P-GP, but the mechanism giving rise to this phenomenon remains unclear.

Considering that P-GP regulates the absorption, distribution and excretion of abundant medicines, the alterations of P-GP expression and function under the vitamin A deficiency condition may lead to significant alterations in the distribution of drugs, bringing about changes of toxicological or pharmacological activity. For example, pharmacological activities of phenobarbital on CNS [39] and the second-generation of Hi-receptor antagonists [40] were reported to be enhanced in diabetic mice. As vincristine is a typical anti-tumor drug, as well as a P-GP substrate, we chose it as an indicator to investigate the alterations of tissue distribution in vitamin A-deficient rats. The results (Table 3) showed that tissue-to-plasma concentration ratios were significantly increased in the indicated tissues of vitamin A-deficient rats. This indicated that if patients under a vitamin A deficiency condition were given the same dosage of drugs as the normal population, this might cause a serious toxic reaction and tissue damage.

It is also known that besides medical agents, P-GP also transports some intrinsic substrates, like steroid hormones and β-amyloid [41,42], and that P-GP may regulate ion channel activity via direct interaction, suggesting that functional alteration of P-GP has a significant impact on the physiological environment [43]. Therefore, whether the alterations of P-GP expression and function may affect the distributions of endogenous substances in the vitamin A deficiency population needs further investigation.

In summary, our present study demonstrates that P-GP expression and function may be altered in cerebral cortex, hippocampus, liver and kidney of rats under the vitamin A deficiency condition, and the alterations may cause changes to the tissue distribution of drugs. These results provide useful information for elucidating the drug dosage and interaction, as well as the pharmacokinetic variability in the vitamin A-deficient population.
